# The pro-atherogenic effects of macrophages are reduced upon formation of a complex between C-reactive protein and lysophosphatidylcholine

**DOI:** 10.1186/1476-9255-9-42

**Published:** 2012-10-31

**Authors:** Mi-Kyung Chang, Karsten Hartvigsen, Jewon Ryu, Yuna Kim, Ki Hoon Han

**Affiliations:** 1 Department of Medicine, University of California San Diego, La Jolla, CA, USA; 2 University of Ulsan College of Medicine, Asan Medical Center, 388-1 Pungnap-2 dong Songpa-gu 138-736, Seoul, South Korea; 3 Present address: Bayer Korea, 7th fl. Samsung-Boramae Omni Tower, 395-62, Sindaebang dong Dongzak-gu, Seoul, South Korea

**Keywords:** C-reactive protein, Lysophosphatidylcholine, Macrophages, Foam cells

## Abstract

**Rationale:**

C-reactive protein (CRP) and lysophosphatidylcholine (LPC) are phosphorylcholine-(PC)-containing oxidized phospholipids (oxPLs) found in oxidized LDL (oxLDL), which trigger pro-atherogenic activities of macrophages during the process of atherosclerosis. It has been previously reported that CRP binds to the PC head group of oxLDL in a calcium-dependent manner. The aim of this study was to investigate the importance of binding between CRP and LPC to the pro-atherogenic activities of macrophages.

**Objectives and findings:**

A chemiluminescent immunoassay and HPLC showed that human recombinant CRP formed a stable complex with LPC in the presence of calcium. The Kd value of the binding of the CRP-LPC complex to the receptors FcγRIA or FcγRIIA was 3–5 fold lower than that of CRP alone. The CRP-LPC complex triggered less potent generation of reactive oxygen species and less activation of the transcription factors AP-1 and NF-kB by human monocyte-derived macrophages in comparison to CRP or LPC alone. However, CRP did not affect activities driven by components of oxLDL lacking PC, such as upregulation of PPRE, ABCA1, CD36 and PPARγ and the enhancement of cholesterol efflux by human macrophages. The presence of CRP inhibited the association of Dil-labelled oxLDL to human macrophages.

**Conclusions:**

The formation of complexes between CRP and PC-containing oxPLs, such as LPC, suppresses the pro-atherogenic effects of CRP and LPC on macrophages. This effect may in part retard the progression of atherosclerosis.

## Background

The process of atherosclerosis is facilitated by inflammation and oxidative stress in the arterial wall [1]. Levels of C-reactive protein (CRP) are correlated with the level of inflammation and a persistently elevated serum level of CRP is a risk factor for the development of atherosclerotic cardiovascular diseases [2,3]. CRP detected in atherosclerotic plaques may be due to circulating CRP [4] or *de novo* CRP synthesized by macrophages and vascular smooth muscle cells [5]. CRP directly triggers the activation of Fc-gamma receptors (FcγRs) [6] and induces a number of innate immune responses including complement activation, monocyte recruitment, and the expression of cytokines and inflammatory mediators by macrophages [7].

We previously demonstrated that CRP can specifically bind to oxidized LDL (oxLDL) but not to non-oxidized native LDL [8]. We further identified that the phosphorylcholine (PC) head group of oxidized phospholipids (oxPLs) such as oxidized 1-palmitoyl-2-arachidonoyl-*sn*-glycero-3-phosphorylcholine (oxPAPC) and 1-palmitoyl-2-oxovaleroyl-*sn* glycero-3-phosphorylcholine (POVPC) is responsible for binding to CRP [8]. The PC-containing phospholipid lysophosphatidylcholine (LPC) is found in body fluids, including blood and ascites, in a complex with albumin and native LDL particles, where it is important for the transport fatty acids and choline [9]. Oxidation dramatically increases the amount of LPC in LDL particles by more than 10-fold, mainly through the enzymatic modification of PC by LDL-associated phospholipase A2 (PLA2) [10,11]. Like CRP, LPC exists in the atherosclerotic arterial wall [12,13] and triggers a number of pro-atherogenic responses [14].

In the present study, we investigated whether binding between the two potentially atherogenic factors CRP and LPC modulates their activities. We found that the activities of CRP and LPC were suppressed when they formed a complex with each other. Furthermore, co-stimulation of macrophages with both CRP and LPC triggered less potent pro-inflammatory activities and oxidative stress than when they were stimulated by either CRP or LPC alone.

## Methods

### Cell culture and animal care

Human macrophages were prepared from circulating monocytes. Briefly, fresh whole blood was withdrawn from healthy volunteers under a fasting state. Three millilitres of whole blood containing 3 mM EDTA was carefully layered onto Picoll Hypaque (1:1 = v/v, d = 1.077 g/ml, Sigma Chemical Co.) and peripheral blood mononuclear cells were separated by centrifugation (600 g, 22°C, 15 min) [15]. Peripheral blood mononuclear cells were immediately plated into a culture dish and incubated in RPMI medium supplemented with 20% autologous serum and antibiotics such as penicillin (100 units/ml) and streptomycin (100 μg/ml) until experiments were performed. 293FT cells (American Type Culture Collection, Manassas, VA) were maintained in high glucose DMEM supplemented with 10% FBS, penicillin (100 units/ml), and streptomycin (100 μg/ml) in a 5% CO2/37°C incubator.

The human recombinant CRP preparation used in the experiment was confirmed to be free of immunoglobulins and endotoxins [16]. All experiments with CRP were performed in the presence of 25 μg/ml polymyxin B to avoid interference from endotoxins. OxLDL with or without DiI (1,1^′^ - Dioctadecyl - 3,3,3^′^,3^′^ - tetramethylindocarbocyanine iodide) labelling was purchased from INTRACEL, MD, USA.

Human CRP cDNA (GeneBank accession no. NM_000567) was PCR amplified using the following primers, 5^′^-TGAATTCAGGCCCTTGTATC-3^′^(sense) and 5^′^-TCCCAGCATAGTTAACGAGC-3^′^(antisense). The complete nucleotide sequence was cloned into the pcDNA3.1 expression vector (Invitrogen) (CRP-pcDNA3.1) and the sequence was confirmed by direct DNA sequencing. For transfection, 1 μg of CRP-pcDNA3.1 was added to 10^5^ macrophages in Opti-MEM (Gibco-BRL, Grand Island, NY) medium in the presence of Fugene6 agent (Roche) and incubatedfor 6 h. For the promoter assay, 293FT cells at 75% confluency were transfected with PPRE-Luc, pRL-TK or pELAM-Luc-NFkB vector as described above using the Superfect transfection reagent (Qiagen Inc.). The viability of the transfected cells was more than 85% as confirmed by propidium iodide staining. In a subset of experiments, macrophages were pre-incubated with 15 μg/ml of the monoclonal anti-FcγRI (clone:10.1, Invitrogen) and anti-Fcγ RII (clone:7.3, Ancell) IgGs for 30 min.

### Measurement of CRP binding to LPC

CRP binding to LPC was determined using a chemiluminescent immunoassay as described previously [8]. In brief, LPC in PBS containing 0.27 mM EDTA was added to each well of a 96-well, white, round-bottomed microtitration plate (Dynex Technologies, Chantilly, VA) and incubated overnight at 4°C. Nonspecific binding sites were blocked using 2% BSA/PBS and each well was incubated for 1 h with CRP in 1% BSA/buffer A (10 mM TBS/2 mM CaCl_2_/1 mM MgCl_2_, pH 7.4). The wells were washed, and the bound CRP was labelled with a rabbit anti-CRP IgG (Abcam) and alkaline phosphatase-labelled goat anti-rabbit IgG (BioLegend). After incubating for 1–2 h with 50% Lumi-Phos 530, luminescence was determined using a Dynatech luminometer (Dynex Technologies). CRP binding was quantitated as the number of relative light units detected over 100 ms (RLU/100 ms).

The formation of complexes between CRP and LPC was further analysed by an HPLC assay using a Waters-Alliance HPLC system. LPC, CRP or CRP-LPC complexes in PBS were separated using an Apollo Silica 5u (250 mm × 4.6 mm) column (Alltech Associates, Inc.). The solvent used for the analysis was ammonium sulfate (5 mM final conc, pH 6.0) in a solution of 60% hexane, 40% isopropanol (v/v). The flow rate used was 1.5 ml/min, and the absorbance of phospholipids in the samples was measured at 205 nm using a Waters 2487 dual λ absorbance detector. Data were expressed as absorbance units (AU) per running time (minutes).

### The binding of CRP and the CRP-LPC complex to FcγRIA and FcγRIIA

293FT cells were transfected with FcγRIA- or FcγRIIA-pcDNA3.1 vector using Fugene6 as described above. After 24 h, cells were replenished with DMEM medium and incubated with the stated concentration of CRP alone or the CRP-LPC complex for 30 min at room temperature. The cell-bound CRP was labelled with a monoclonal anti-CRP mouse IgG (clone CRP-8, Sigma; 1:200) and a FITC-conjugated goat anti-mouse secondary antibody (10 μg/ml final conc, Jackson Immunoresearch). The amount of cell-associated fluorescence was estimated using flow cytometry. Nonspecific binding of CRP or CRP-LPC complex was estimated by parallel experiments using 293FT cells transfected with an empty vector (mock). Kd values and Bmax values of CRP-binding were calculated using PRISM software.

### Quantification of intracellular reactive oxygen species (ROS)

To estimate the intracellular generation of ROS, human monocyte-derived macrophages were labelled with 5 μM H_2_DCFDA (Molecular Probes) for 30 min at room temperature and subsequently stimulated with 10 μg/ml CRP, LPC, or CRP-LPC complexes for 1 h at 37°C. The intensity of cell-associated fluorescence, which reflects the amount of intracellular ROS, was measured and analyzed using a TCS-SP2 confocal microscopy system (Leica Microsystems, Nussloch GmbH, Germany). Spontaneous H_2_DCFDA photo-oxidation was minimised by imaging cells with a single rapid scan (30 frames/sec for 512x512 pixel). The cell-associated fluorescence, which reflects the amount of intracellular ROS, in the acquired images converted to multi-coloured computerised digital images. The amount of intracellular ROS was represented by different colours where red > yellow > green > white. In order to estimate the intensity of cell-associated fluorescence, at least 100 cells in the same image frame was used for the analysis and three independent experiments were performed in duplicate.

### Electrophoretic mobility shift assay (EMSA)

Activation of AP-1, NF-κB and PPARγ in macrophages was measured by EMSA using [γ-^32^P]-ATP-labelled specific oligonucleotides containing sequences that specifically react with AP-1 (5^′^-CGCTTGATGACTCAGCCGGAA-3^′^), NF-κB (5^′^-AGTTGAGGGGACTTTCCCAGGC-3^′^), and PPAR (5^′^-CAAACTAGGTCAAAGGTCA-3^′^) protein, respectively (Santa Cruz Biotechnology Inc.) [17]. To ensure the bands were specific, a cold reaction was simultaneously performed with a 25-fold excess of unlabelled oligonucleotides.

### Promoter assay

293FT cells were transfected with PPRE-Luc pRL-TK or pELAM-Luc-NFkB. CRP, LPC or CRP-LPC complexes were added 4 h after transfection, and a luciferase assay was performed 24 h later using a commercial kit (Promega, Madison, WI). , A promotor assay was performed under identical conditions using 293FT cells transfected with pHook^TM^-β-gal (Invitrogen) in order to estimate the efficacy of transfection. Obtained results were normalized relative to the amount of cellular protein (Bio-Rad protein assay kit) and β-gal activities, and expressed as luciferase activities derived from untreated transfected cells.

### Cholesterol efflux

Human monocyte-derived macrophages (2000 cells per 24-well plate) were incubated with 2 mCi/mL [^3^H]cholesterol for 48 hours. Cells were washed 2 times with PBS and further incubated for 4 hours in the presence of 2.5% freshly-isolated human serum. An aliquot of medium and cell lysate was counted for [^3^H]cholesterol radioactivity using liquid scintillation analyzer (Packard). Percent of cell-associated [^3^H]cholesterol released to medium via the process of efflux were calculated as follows; [^3^H]cholesterol_medium_/([^3^H]cholesterol_medium_ + [^3^H]cholesterol _cell lysate_) × 100, and normalized to cell protein. Binding and uptake of DiI-labelled oxLDL by macrophages.

oxLDL labelled with the lipophilic fluorescent dye DiI in accordance with manufacturer’s instructions, which can be detected at the emission wavelength of 570 nm, was obtained from Biomedical Technologies, Inc. Human macrophages were plated at a density of 1×10^5^ cells per well in a 12 well plate,washed twice with RPMI 1640 medium, and then incubated with 25 μg/ml DiI-labelled oxLDL (DiI-oxLDL) in the presence or absence of CRP (1–25 μg/ml) for 30 min in a 5% CO_2_/37°C incubator. In a subset of experiment, FcγRs were pre-blocked with 15 μg/ml of the monoclonal anti-FcγRI (clone:10.1, Invitrogen) and anti-Fcγ RII (clone:7.3, Ancell) mouse antibodies at 37°C for 15 min prior to the incubation with DiI-oxLDL. The intensity of cell-associated fluorescence was analysed by flow cytometry using CELLQUEST software.

### Measurement of the expression levels of CRP and cytokines

The level of MCP-1, MMP-1, MMP-9, IL-1β, and IL-8 mRNA was measured by semi-quantitative and real time PCR. Specific sequences were amplified using the primer sets and number of cycles (94°C for 30 s, 60°C for 1 min and 72°C for 1 min) shown in Table 1. PCR products were electrophoresed on ethidium bromide-containing agarose gels and the band intensity was measured by densitometric scanning. The linearity of the amplification was established using serial dilutions of template DNA. Real time PCR was performed with SYBR Green I using the specific primers shown in Table 1. As an internal standard, GAPDH mRNA was amplified and analysed under identical conditions using a pair of specific primers described in Table 1. The Ct value (the cycle number at which emitted fluorescence exceeded an automatically determined threshold) for target cDNA was corrected by the Ct value for GAPDH and expressed as ΔCt. Data are expressed as fold changes in the amount of mRNA, which was calculated as follows; (fold changes) = 2^(ΔCt for untreated cells - ΔCt for treated cells)^.

**Table 1:** **PCR primers and reactionconditions**

**Gene**	**Gene accession number**	**Primers**	**Product size**
IL-1β	NM_000576.2	5^′^-AGTGGTGTTCTCCATGTCCT-3^′^ (sense)	398 bp
		5^′^-AGTCAGTTATATCCTGGCCG-3^′^ (antisense)	
IL-8	NM_000584.3	5^′^-CTTTCAGAGACAGCAGAGCA-3^′^ (sense)	510 bp
		5^′^-CCTACAACAGACCCACACAA-3^′^ (antisense)	
MCP-1	NM_002981.1	5^′^-CACTCACCTGCTGCTACTCATT-3^′^ (sense)	807 bp
		5^′^-TGTTGAACCAGGATTCACAGAG-3^′^ (antisense)	
MMP-1	NM_002421.3	5^′^-CCATTCTACTGATATCGGGG-3^′^ (sense)	451 bp
		5^′^-GCCAAAGGAGCTGTAGATGT-3^′^ (antisense)	
MMP-9	NM_004994.2	5^′^-GAGATTGGGAACCAGCTGTA-3^′^ (sense)	569 bp
		5^′^-TGCAGGATGTCATAGGTCAC-3^′^ (antisense)	
GAPDH	NM_002046.4	5^′^-GACCCCTTCATTGACCTC-3^′^ (sense)	360 bp
		5^′^-GCTAAGCAGTTGGTGGTG-3^′^ (antisense)	

### Immunoblotting assay

Twenty micrograms of total 293FT cell lysate was separated by electrophoresis (300 mA for 2 h) on a 10% SDS-PAGE gel and transferred onto an Immobilon PVDF membrane (Millipore Corporation, Bedford, MA). Blots were blocked with 5% skimmed milk in TBS with 0.01% Tween-20 (TBS-T) for 60 min and incubated overnight at 4°C with the following antibodies; mouse monoclonal anti-CD36 (Clon: SMO; Ancell corporation, MN USA), mouse monoclonal anti-ABCA1 (AB.H10; Abcam corporation Cambridge UK), mouse monoclonal anti-PPARγ (Santa Cruz biotechnology. INC) and mouse monoclonal anti-α-actin (Sigma Chemical Co. St. Louis, MO). The membrane was washed in TBS-T for 10 min three times and incubated with goat anti-mouse HRP (1:10000, Jackson Immuno Research Lab Inc) for 60 min at room temperature. After washing with TBS-T for 10 min three times, the membrane was developed by a chemiluminescent detection kit (ECL-kit, Amersham, Piscataway, NJ). The amount of signal generated was quantified by scanning photo-densitometry using a MULTI-IMAGE analysis system and Quantitation software (Bio-Rad Laboratories Inc., Hercules, CA).

### Statistical analysis

SPSS was used to perform statistical analysis. Differences between two groups were determined by unpaired Student *t* test. Differences between multiple groups were determined by two-way analysis of variance (ANOVA), where appropriate. Differences were considered significant when p < 0.05. The binding of the CRP-LPC complex to FcγRIA and IIA was analysed using PRISM 3.0 software.

## Results

### CRP forms a complex with LPC in a calcium-dependent manner

**Figure 1:**
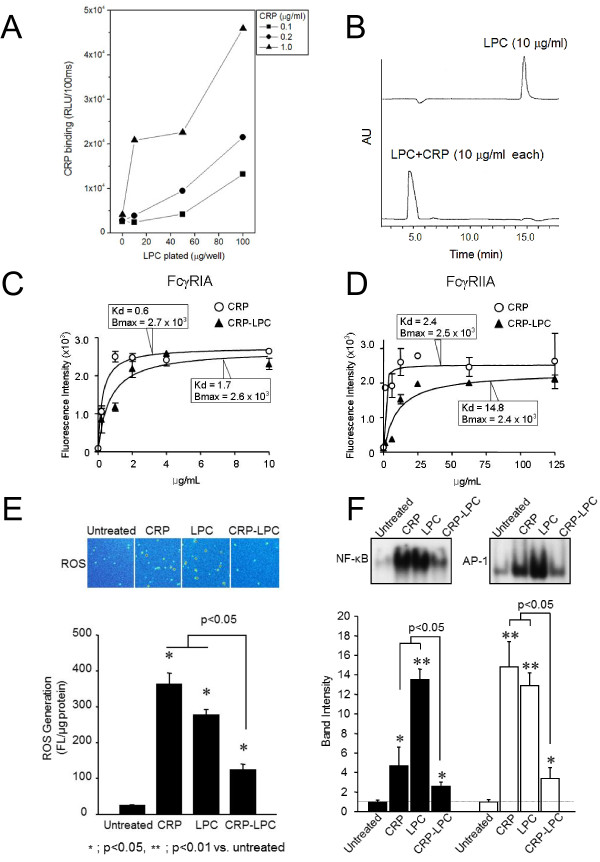
**Complex formation between CRPand LPC and theimpact of the CRP-LPCcomplex on FcγRs bindingkinetics and macrophage activities.** (**A**). CRP bound to LPC-coated plate was detected with a specific anti-CRP antibody and alkaline phosphatase-labelled secondary antibody. Data were expressed as relative light units over 100 ms (RLU/100 ms). The figure represents one of three independent experiments. (**B**). LPC in the presence or absence of CRP (10 μg/ml each in the presence of 2 mM calcium) was detected by HPLC using an Apollo Silica 5u column. Data are expressed as absorbance units (AU) at 205 nm over time. The figure represents one of three independent experiments. (**C** and **D**). The specific binding of CRP or CRP-LPC (up to 10 μg/ml) to 293FT cells transfected with FcγRIA (C) or FcγRIIA (D) was obtained as described in the Methods section. Data shown are mean values ± S.D. from three independent experiments. Kd and Bmax values were analysed using Prism software. (**E**). H_2_DCFDA-labelled human macrophages were stimulated with 10 μg/ml CRP, LPC or CRP-LPC complexes for 30 min and the generated intracellular ROS (red > yellow > green > white in the upper panel) was detected by confocal microscopy. Data represent mean values ± S.D. of the amount of intracellular ROS from three independent experiments. (**F**). NF-kB and AP-1 activity of human macrophages treated with 10 μg/mL CRP, LPC, or CRP-LPC complexes were measured by EMSA. The EMSA image represents one of three independent experiments. Data represent mean values ± S.D. from three independent experiments.

A chemiluminescent immunoassay showed that CRP bound to LPC in a dose-dependent manner (Figure 1A, p < 0.01 ANOVA). The CRP binding to LPC was calcium-dependent, as binding was abolished by calcium depletion or the addition of EDTA (data not shown). HPLC showed that the peak representing LPC gradually disappeared as CRP was added, while a new peak was detected at a different position, suggesting LPC forms a complex with CRP. The LPC peak at the original position became undetectable when CRP was added at a ratio of 1:1(w:w) and this ratio was used for subsequent experiments in this study (Figure 1B). HPLC confirmed that the CRP-LPC complex was stable for at least 48 h at 37°C (data not shown).

### The CRP-LPC complex binds less efficiently to FcγRIA and FcγRIIA in comparison to CRP alone

FcγRIA or FcγRIIA was expressed in 293FT cells, the CRP-LPC complex was added, and the level of binding of the complex to cells was estimated by flow cytometry analysis. The Kd values of CRP-LPC complex binding to FcγRIA or FcγRIIA were 3–5 times higher in comparison to CRP alone, indicating binding of CRP to LPC lowers the binding affinity of CRP for FcγRs (Figure 1C and 1D). However, the maximal binding of the CRP-LPC complex to either FcγRIA- or FcγRIIA, as estimated by Bmax values, was comparable to that of CRP alone.

### The CRP-LPC complex induces less potent intracellular ROS production and less activation of AP-1 and NF-kB

Confocal microscopic images showed that H_2_DCFDA-labelled macrophages derived from human monocytes displayed cell-associated fluorescence after treatment with CRP or LPC alone for 30 min, indicating the generation of ROS. The level of intracellular ROS produced by the CRP-LPC complex was less potent (Figure 1E). The activation of AP-1 and NF-kB by CRP or LPC was confirmed by EMSA. As observed for ROS generation, the level of AP-1 and NF-kB activation induced by the CRP-LPC complex was significantly lower than that induced by CRP or LPC alone (Figure 1F).

### Binding of CRP and LPC reduces the production of pro-inflammatory mediators by human macrophages

Real time PCR showed that both CRP and LPC stimulated expression of MMP-1, MMP-9, MCP-1, IL-1β, and IL-8 by human macrophages after 24 h (Table 2). CRP induced expression of MMP-1 from an undetectable basal level and this effect was suppressed by the addition of LPC. Similarly, the induction of MMP-9, MCP-1, IL-1β and IL-8 by LPC was antagonized by CRP.

**Table 2:** **Changes in the mRNAexpression levels of MMP-1,MMP-9, MCP-1, IL-1β andIL-8 in response tothe CRP-LPC complex**

**mRNA**	**Untreated**	**CRP**	**LPC**	**CRP-LPC**
**Concentration**		**10 μg/ml**	**10 μg/ml**	**10 μg/ml each**
**ΔCt of average±S.D.**				
MMP-1	ND	10.63±0.76	9.78±3.26	13.63±1.08 ^┼^
		(NC)	(NC)	(NC)
MMP-9	4.82±0.19	3.50±0.34*	3.47±0.28*	5.10±0.26 ^┼^
		(2.6)	(2.5)	(0.8)
MCP-1	4.38±0.06	1.40±0.08*	2.18±0.02*	2.95±0.01*^┼^
		(7.9)	(4.6)	(2.7)
IL-1β	12.04±0.29	1.63±0.01*	10.46±0.29*	2.38±0.21*^┼^
		(3258.5)	(3.0)	(809.0)
IL-8	9.34±0.08	0.73±0.04*	7.83±0.02*	3.02±0.48*^┼^
		(389.4)	(2.9)	(79.6)

Human macrophages were incubated with 10 μg/ml CRP, LPC or CRP-LPC for 24 h and mRNA expression levels of MMP-1, MMP-9, MCP-1, TNF-α, IL-1β, IL-8 and GAPDH were estimated by real-time PCR. Data analyzed were from three independent experiments. Fold changes in mRNA levels are presented, which were calculated from ΔCt values as described in the Methods section.

Each PCR was performed with 40 amplification cycles. *;p < 0.05 vs. Untreated macrophages. ^┼^; p < 0.05 vs. CRP and LPC treated macrophages. Significance values were calculated using a the unpaired *t* test. ND; not detectable within 40 cycles of amplification, NC; non-calculable.

### CRP does not suppress PPRE activation induced by oxidized linoleic acid (oxLA) lacking PC

**Figure 2:**
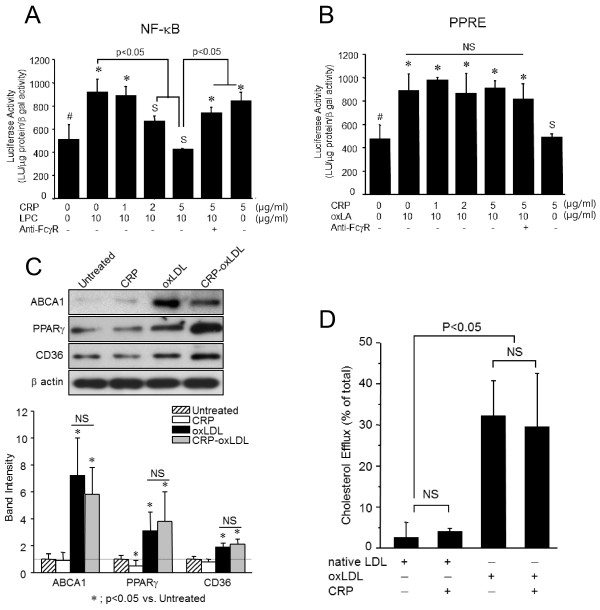
**CRP sustains oxLDL-stimulated expressionof ABCA1, PPARγ, CD36and PPRE leading tocholesterol efflux.** (**A** and **B**). 293FT cells transfected with pELAM-Luc-NFκB-Luc pRL-TK (**A**) or pELAM-Luc-PPRE-Luc pRL-TK (**B**) were treated with hCRP (up to 5 μg/ml/24h), LPC or oxLA (10 μg/ml/24h). FcγRs were blocked with monoclonal neutralizing antibodies against FcγRI (clone:10.1) and FcγRII (clone:7.3) (‘Anti-FcγR’, 15 μg/ml each). Data represent mean values ± SD of luciferase activity of the cell lysates after adjusting relative to the β-gal activity and the protein content. *; p < 0.05 vs the control (#), S; not statistically significant vs. the control (#), NS; not statistically significant each other. (**C**). Human macrophages were treated with 10 μg/ml/24h hCRP, fully oxidized LDL (oxLDL), or a mixture of hCRP-oxLDL, and the protein expression levels of ABCA1, PPARγ, and CD36 were estimated by an immunoblot assay. The immunoblotting image represents one of three independent experiments. Data are given as mean ± SD from three independent experiments. *; p < 0.05 vs. untreated, NS; not statistically significant. (**D**). Human macrophages were incubated with 2 mCi/mL [^3^H]cholesterol for 24 h, and 10 μg/ml oxLDL or hCRP was added and maintained further for 24 h. Unbound [^3^H]cholesterol was washed off and cells were further incubated for 4 h. The percentage of cell-associated [^3^H]-cholesterol released into the media in 4 h was obtained by performing a cholesterol efflux assay as described in the Methods. Data are given as mean ± SD from three independent experiments. NS; not statistically significant. CRP does not inhibit oxLDL-stimulated cholesterol efflux by human macrophages.

A promoter assay was performed using FcγRIA(+) 293FT cells, which clearly showed that both CRP and LPC induced NF-κB activation (Figure 2A). As expected, CRP inhibited LPC-induced NF-κB activation in a dose-dependent manner (p < 0.01, ANOVA). We confirmed that oxLA, an oxidized moiety in oxLDL which lacks PC, induced PPRE activation. CRP did not activate PPRE nor did it affect PPRE activation induced by oxLA (Figure 2B, p > 0.05 ANOVA).

The degree of lipid accumulation in macrophages can also be determined by the efficacy of cholesterol efflux. oxLDL upregulated the expression level of genes that promote cholesterol efflux; e.g., ABCA1, PPARγ, and CD36 (Figure 2C). As expected following the upregulation of these genes, oxLDL dramatically increased cholesterol efflux by human macrophages from 2.6±3.7% to 32.2±8.6% (p < 0.05) and the presence of CRP did not significantly affect oxLDL-stimulated cholesterol efflux (Figure 2D).

### CRP inhibits foam cell formation in vivo and oxLDL uptake by human macrophages

**Figure 3:**
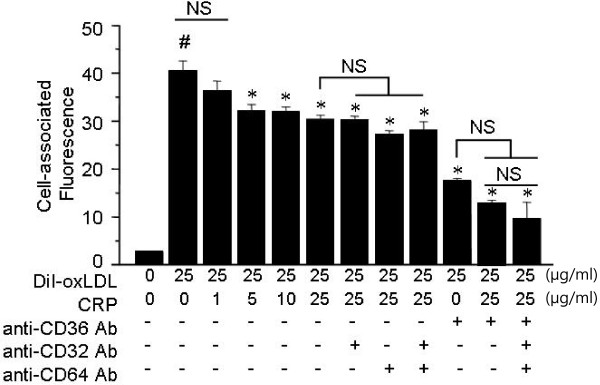
**Reduced foam cell formationin human CRP transgenicmice may be explainedby CRP inhibition ofthe association of oxLDLwith macrophages.** Human macrophages were incubated with 25 μg/ml of DiI-oxLDL for 30 min with or without hCRP as indicated. In a subset experiments, FcγRI (CD64), FcγRII (CD32) or CD36 was pre-blocked by incubating with 15 μg/mL monoclonal IgGs for 15 min prior to incubation with DiI-oxLDL. The data represent mean ± SD of specific cell-associated fluorescence from three independent experiments. Significance was determined using an ANOVA followed by unpaired *t* tests. *; p < 0.05 vs. the control (#), NS; not statistically significant.

We studied whether CRP affects oxLDL uptake by human macrophages. Flow cytometry showed that the association of DiI-oxLDL with human macrophages was mediated predominantly through the scavenger receptor CD36, as an anti-CD36 antibody could inhibit >60% of the cell-associated fluorescence (Figure 3). CRP inhibited the association of DiI-oxLDL to macrophages in a dose-dependent manner. CRP significantly inhibited >30% of the CD36-dependent oxLDL uptake (Figure 3; p < 0.05, ANOVA). Unlike CD36, the functional blocking of FcγRI (CD64) and FcγRII (CD32) did not affect the association of DiI-oxLDL with human macrophages regardless of the presence of CRP (Figure 3).

## Discussion

In this study we have shown that CRP forms a stable complex with LPC, a major PC-containing phospholipid in oxLDL, supporting our previous observations that exposure of the PC head group of unsaturated fatty acids following oxidation is a prerequisite for binding to CRP [8]. Previous studies have described how two Ca^2+^ binding sites in the CRP pentamer [18] directly bind to two oxygen atoms within a phosphate group of PC [19], while specific binding sites for FcγRs are located on the opposite plane of CRP [20]. Electron microscopy studies have demonstrated that binding of CPR to PC-containing ligands, including LPC, induces the partial dissociation of the pentameric structure of CRP to an open-ring-like structure [21]. Results of our binding assay clearly showed binding between CRP and LPC decreases their binding affinity to FcγRI and II without changing their maximal binding capacity. Therefore, it is possible that CRP-bound LPC may not directly interfere with binding of CRP pentamers to FcγRs. Instead, conformational changes in the LPC-bound CRP pentamer may distort binding sites for FcγRs, resulting in less efficient binding of the CRP-LPC complex to FcγRs. This may lead to less potent activation of FcγRs, thus reducing NF-kB activation [22] and the generation of ROS [23], as observed in this study.

The formation of atherogenic foam cells is stimulated by defects in cholesterol efflux. Macrophages regulate cholesterol efflux through uptake of oxLDL by CD36 and release of oxLDL by ABCA1. In this study, oxLDL profoundly upregulated the expression of CD36 and ABCA1 in macrophages, and enhanced cholesterol efflux through PPARγ activation as previously described [24]. Interestingly, we found that the presence of CRP did not affect oxLDL-stimulated cholesterol efflux or modify regulation of the related genes. This observation suggests that CRP does not affect the activities of moieties lacking PC in oxLDL, such as oxysterol-stimulated LXR expression and/or oxidized fatty acid-induced PPARγ upregulation [25], which promote cholesterol efflux.

Our hypothesis was further confirmed using a promoter assay, in which CRP blocked NF-κB activation by PC-containing LPC, while PPRE activation induced by PC-lacking oxLA was unaffected. Moreover, the in vitro experiments showed that CRP interferes with the binding of oxLDL to macrophage scavenger receptors such as CD36. We previously obtained similar findings showing that anti-PC antibody (EO6) binds to the PC-epitope in oxLDL and inhibits the binding of oxLDL to macrophages [26]. A previous study also showed that complexes of CRP and enzymatically-modified LDL (E-LDL) induced less effective foam cell formation when compared to E-LDL alone [27]. However, several other studies are contradictory to ours, and report that the presence of CRP may enhance the uptake of oxLDL through FcγRs. In these studies, the U937 human leukemic monocytic cell line representing undifferentiated form of monocytes [28] or monocytes freshly-recruited to inflammatory foci [29] were used. In this study, we used fully-matured human macrophages that had a 12–20 fold higher expression of CD36, in comparison to immature monocytes, which mediates 40–50% of oxLDL binding [30]. We found that the uptake of oxLDL was mostly through CD36, and that FcγR-dependent trafficking of CRP-oxLDL complexes was minimal. A previous study showed that the proteasome activity of intra-plaque macrophages in hCRP/ApoB^100/100^/LDLR^−/−^ mice was enhanced by CRP [31], which resulted in degradation of oxidized and misfolded proteins and reduced oxidative stress. Taken together with our findings, it is possible that CRP suppresses the transformation of macrophages into foam cells via three distinct mechanisms; interference of oxLDL uptake, degradation of oxidized biomolecules, and maintenance of active cholesterol efflux.

Our observations do not contradict the current model that CRP and oxLDL are pro-atherogenic mediators for macrophages. This study demonstrates that either LPC, a PC-containing moiety in oxLDL, or CRP alone, activate NF-kB, and stimulate the generation of ROS, as well as pro-inflammatory mediators and cytokines in human macrophages [32-34]. Additionally, the study suggests a potential mechanism in which complex formation between CRP and PC-epitopes in oxLDL may alleviate oxidative and pro-inflammatory stress in macrophages, which could explain the findings of a number of previous studies. It has been shown that CRP forms a complex with E-LDL and inhibits E-LDL-induced formation of the membrane attack complex [35]. Moreover, the binding of CRP to apoptotic cells suppressed CRP-induced complement activation, while the production of tumour growth factor alpha, an anti-inflammatory cytokine, by apoptotic cells was unaffected [36].

## Conclusions

In early and advanced stages of human atherosclerotic plaques, both CRP and oxLDL are co-localized with macrophages [37,38]. It is possible that the functional inhibition of CRP through complex formation with PC-containing phospholipids and vice versa may not only retard the morphological change of macrophages into foam cells but also suppress the pro-inflammatory and oxidative activities of macrophages that can be triggered by either CRP or oxLDL alone.

## Competing interests

The authors declare that they have no competing interests.

## Authors’ contributions

M-KC, Contributed to the conception and design of the study and to the acquisition, analysis and interpretation of data.KH, Contributed to preparation of the manuscript. JR, Contributed to the conception and design of the study and to the acquisition, analysis and interpretation of data. YK, Contributed to the conception and design of the study and to the acquisition, analysis and interpretation of data. KHH. Contributed to the conception and design of the study, to the acquisition, analysis and interpretation of data and to preparation of the manuscript. All authors read and approved the final manuscript.
